# The slowdown of Y chromosome expansion in dioecious *Silene latifolia* due to DNA loss and male-specific silencing of retrotransposons

**DOI:** 10.1186/s12864-018-4547-7

**Published:** 2018-02-20

**Authors:** Janka Puterova, Zdenek Kubat, Eduard Kejnovsky, Wojciech Jesionek, Jana Cizkova, Boris Vyskot, Roman Hobza

**Affiliations:** 1 Department of Plant Developmental Genetics, Institute of Biophysics, Czech Academy of Sciences, Kralovopolska 135, 612 00 Brno, Czech Republic; 2 Department of Information Systems, Faculty of Information Technology, Brno University of Technology, 61200 Brno, Czech Republic; 3 Centre of Plant Structural and Functional Genomics, Institute of Experimental Botany, Czech Academy of Sciences, 783 71 Olomouc - Holice, Czech Republic

**Keywords:** Epigenetics, Genome size, *Silene latifolia*, Transposable elements, Y chromosome

## Abstract

**Background:**

The rise and fall of the Y chromosome was demonstrated in animals but plants often possess the large evolutionarily young Y chromosome that is thought has expanded recently. Break-even points dividing expansion and shrinkage phase of plant Y chromosome evolution are still to be determined. To assess the size dynamics of the Y chromosome, we studied intraspecific genome size variation and genome composition of male and female individuals in a dioecious plant *Silene latifolia*, a well-established model for sex-chromosomes evolution.

**Results:**

Our genome size data are the first to demonstrate that regardless of intraspecific genome size variation, Y chromosome has retained its size in *S. latifolia*. Bioinformatics study of genome composition showed that constancy of Y chromosome size was caused by Y chromosome DNA loss and the female-specific proliferation of recently active dominant retrotransposons. We show that several families of retrotransposons have contributed to genome size variation but not to Y chromosome size change.

**Conclusions:**

Our results suggest that the large Y chromosome of *S. latifolia* has slowed down or stopped its expansion. Female-specific proliferation of retrotransposons, enlarging the genome with exception of the Y chromosome, was probably caused by silencing of highly active retrotransposons in males and represents an adaptive mechanism to suppress degenerative processes in the haploid stage. Sex specific silencing of transposons might be widespread in plants but hidden in traditional hermaphroditic model plants.

**Electronic supplementary material:**

The online version of this article (10.1186/s12864-018-4547-7) contains supplementary material, which is available to authorized users.

## Background

Sex chromosomes evolved independently in plants and animals from a pair of ordinary autosomes. Contrary to animals, only 19 plant species possess well-established sex chromosomes. Most of these species bear large Y chromosomes, suggesting an early expanding stage of sex chromosome evolution [1]. Expansion of mainly non-recombining parts of sex chromosomes is frequently accompanied by accumulation of repetitive sequences. This often results in significant genome size variation among closely related dioecious and non-dioecious (gynodioecious, hermaphroditic) species as was shown in *Silene* [2] and *Asparagus* [3]. Out of all repeats, major contributors to genome size variation present transposable elements (TEs). TEs have been reported as players in sex chromosome size dynamics not only in species with established heteromorphic sex chromosomes such as *Silene latifolia* [4], *Rumex acetosa* [5] and *Coccinia grandis* [6] but also participate in the evolution of the young homomorphic sex chromosome system in *Carica papaya* [7].

*S. latifolia* (white campion) possesses a well-established sex determination system with the dominant Y chromosome in males. Contrary to the evolutionary old sex chromosomes in humans, *S. latifolia* sex chromosomes evolved relatively recently, ca. 6 mya [8]. The nuclear genome of *S. latifolia* is arranged in 11 autosomal pairs and one pair of sex chromosomes. The Y chromosome in *S. latifolia* is the largest chromosome in the entire genome, approximately 1.4 times larger than the X chromosome [9]. Although the *S. latifolia* Y chromosome is not heterochromatinised; it has accumulated a significant number of DNA repeats. It was shown that chloroplast and mitochondrial DNA sequences have been transferred on sex chromosomes in *S. latifolia* [10]. Moreover, some microsatellites [11] and satellites [12, 13] are specifically distributed or accumulated on the Y chromosome in this species. A global survey of all the major types of repeats shows that two antagonistic processes - repeat accumulation and repeat spread suppression - form the Y chromosome in *S. latifola* [8].

Here we compare the global genome composition of several *S. latifolia* ecotypes. We focus on differences in genome size dynamics among the ecotypes at the autosomal and sex chromosome level. We address the following questions: How much the Y chromosome varies among *S. latifolia* populations? Does this variation correlate with genome size? Is the Y chromosome still expanding in *S. latifolia*? Which repetitive elements dominantly contribute to Y chromosome expansion in *S. latifolia*? Are these repetitive elements also the main contributors to genome size expansion?

## Methods

### Biological material and genome size estimation

*S. latifolia* seeds of each sex were collected from wild populations across Europe at seven geographical locations (Additional file 1, Additional file 2: Table S1). *S. latifolia* is not protected or endangered species in European countries. Collection of *S. latifolia* seeds comply with national and international guidelines and no permissions were needed. Seeds for all investigated plants were archived and are available upon request at the Institute of Biophysics, Department of Plant Developmental Genetics, Brno, Czech Republic. Plants were grown under greenhouse conditions. Three male and three female individuals were analyzed for each *S. latifolia* accession, and each individual was measured three times on three different days. Nuclear genome size was estimated using flow cytometry according to [14]. Genome size (2C value) was determined considering 1 pg DNA is equal to 0.978 × 10^9^ bp [15] and average genome size of samples from distinct populations is available in Additional file 2: Table S2.

### Processing of whole genome sequencing data

The *S. latifolia* genomes were sequenced by Illumina Nextera MiSeq platform using paired-end protocol. For detailed information about sequencing libraries of individual samples see Additional file 2: Table S3. Raw reads were examined and filtered by quality using FastQC [16] and Trimmomatic tool [17]. All 14 datasets were randomly sampled to represent approximately 0.015×/1C (the exact number of reads is shown in Additional file 2: Table S4) and 3,479,090 reads were analyzed altogether. RepeatExplorer pipeline [18, 19] was used for de novo repeat identification. Resulting clusters were characterized based on similarity searches against RepeatMasker libraries, user custom libraries, in blastn and blastx [20]. Reference sequences of main LTR retrotransposon subfamilies presenting in *S. latifolia* genome were collected using assembled contigs published in [21]. Contigs of these LTR retrotransposons were used as queries for megablast [22] searches against nr/nt database with default settings. For significant hits with GenBank database see Additional file 3. In case of significant hits with unannotated GenBank sequences or no hits, contigs were further searched for the presence of protein domains using CD-Search [23] with default settings. Annotated contigs were used as queries to search for similarities against assembled *S. latifolia* bacterial artificial chromosome (BAC) clones using Geneious 8.1.7 software (http://www.geneious.com, [24]), with similarity threshold set to 80%. Full length genomic copies from BACs were manually annotated in Geneious 8.1.7 and aligned using MAFFT v7.017 [25].

### TE abundance and copy number estimation

To estimate approximate abundance and copy number of main LTR retrotransposon subfamilies in *S. latifolia*, genomic reads were uniquely mapped onto reference sequences of individual subfamilies using Bowtie 2 v2.3.0 [26]. Coverage of subfamilies was obtained by samtools tool [27] using bedcov utility and copy number for the whole genome was calculated using a formula: (subfamily coverage [bp]/subfamily_length [bp])*(100/0.75), where 0.75 represents 0.75% 1C coverage. Density of OgreCL5 subfamily in X chromosomes in comparison to autosomes was estimated according to formula ((F-M)/F)*2/0.15, where *F* is a copy number of OgreCL5 subfamily in female (2n), *M* is a copy number of OgreCL5 subfamily in male (2n) and 0.15 accounts for genome length of X chromosome [9]. To display changes in copy number of individual LTR retrotransposons subfamilies in ecotypes, a difference between male and female copy number was calculated and illustrated using heatmap (see Additional file 4).

### Fluorescence in situ hybridization

Fluorescence in situ hybridization experiments were performed according to [9] with slight modifications. Primers for probe preparation were designed on LTR and GAG or ORF region of selected LTR retrotransposons using Primer3 [28] and are available in Additional file 5. To distinguish Y chromosome arms, X43.1. tandem repeat hybridizing only on the q arm of the Y chromosome has been used [29]. All the above-mentioned procedures and methods were conducted as thoroughly described in Additional file 6.

## Results

### Genome size varies more than Y chromosome size in *S. latifolia* ecotypes

**Fig. 1:**
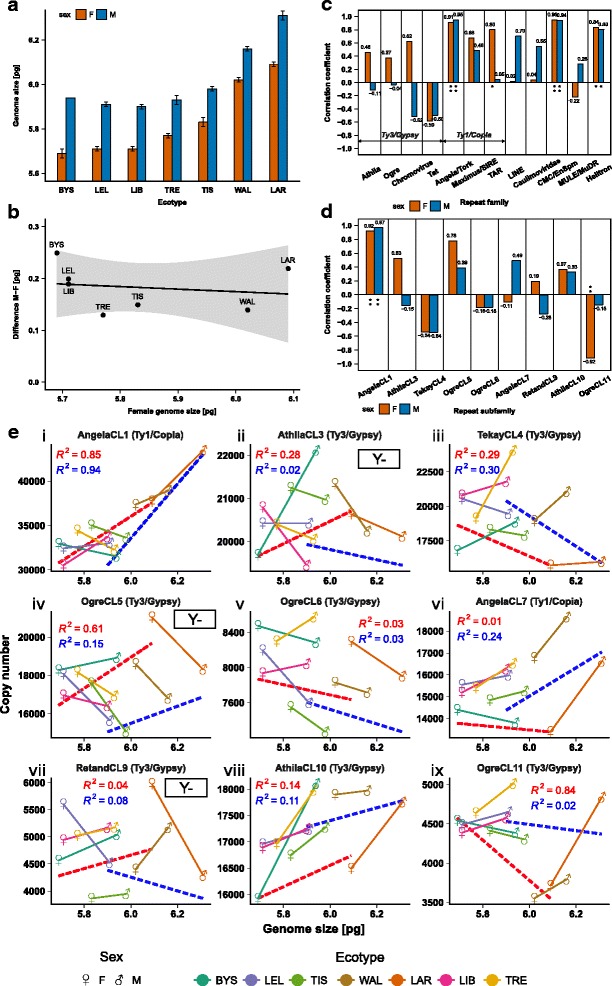
Genome size and composition of *Silene latifolia* ecotypes. **a** Genome sizes of *S. latifolia* male and female genome from eight distinct ecotypes measured by flow-cytometry. Genome size varies from 5.90 pg (LIB) to 6.31 pg (LAR) in males and 5.69 pg (BYS) to 6.09 pg (LAR) in females. Error bars represent SEM. **b** Difference in genome size between sexes caused by Y chromosome. Difference was calculated using a formula: *(M-F)/F*, where *M* corresponds to male genome size and *F* to female genome size. It varies between 2.24% (WAL) and 4.32% (BYS). Black line represents linear regression line of plotted data. Grey area displays 95% confidence interval. **c** Correlation between abundance of repeat families and genome size of both sexes in *S. latifolia*. Correlation coefficient represents Pearson correlation coefficient, n (number of samples) = 7, degrees of freedom = 5. **d** Correlation between abundance of main LTR retrotransposon subfamilies and genome size of both sexes in *S. latifolia*. Correlation coefficient represents Pearson correlation coefficient, n (number of samples) = 7, degrees of freedom = 5. **e** Detailed contribution (copy number vs. genome size) of main LTR retrotransposons to genome size in both sexes. Dashed lines correspond to linear regression between female genome size and element’s copy number (red), and male genome size and element’s copy number (blue). R^2^ represents coefficient of determination (square of the Pearson correlation coefficient), n (number of samples) = 7, degrees of freedom = 5

In order to assess possible intraspecific genome and Y chromosome size variation in *S. latifolia*, male and female genome size in seven distinct ecotypes from central and southern Europe was measured using flow cytometry. Map with the locations of sample collection is depicted in Additional file 1. As shown in Fig. 1a, genome size varies substantially among ecotypes and is always larger in males than females. Male genome sizes vary between 5.90 ± 0.01 pg/2C and 6.31 ± 0.02 pg/2C while female genomes are in the range 5.69 ± 0.02 pg/2C and 6.09 ± 0.01 pg/2C representing 1.07-fold variation in genome size. The excessiveness of male genomes over female genomes (Fig. 1a) reflects the enormous size of the Y chromosome, which is approximately 1.4 times larger than the X [9]. Nevertheless, the proportion of the Y chromosome tends to be in negative correlation with whole genome size (Fig. 1b) which indicates that genome size variation among *S. latifolia* ecotypes is caused predominantly by processes taking place on autosomes and X chromosomes.

### Genome composition

To decipher how individual repeat types contribute to genome size, whole genome shotgun sequencing was performed on males and females of seven ecotypes using Illumina MiSeq platform generating raw 300 bp long paired-end reads. The reads were analyzed by RepeatExplorer [18, 19] as specified in Materials and Methods. The global repeat composition is summarized in Table 1. LTR (Long Terminal Repeat) retrotransposons represented the major fraction of all analyzed genomes, comprising of up to 70% of nuclear DNA. They were mostly represented by *Ty3/Gypsy*-like elements (~ 50%), while *Ty1/Copia*-like elements represented roughly 20% in all genomes. Non-LTR retrotransposons and DNA transposons were much less abundant and occupied ~ 0.3 and ~ 3.3% of genomes, respectively. Tandem repeats formed clusters with a small number of reads in our analysis, and thus they might not present a significant portion of studied genomes.

**Table 1:** Transposable element composition of *Silene latifolia* genome

Classification			Genome proportion [%]													
Repeat Type	Superfamily	Family	BYS-fe	BYS-ma	LEL-fe	LEL-ma	LIB-fe	LIB-ma	TRE-fe	TRE-ma	TIS-fe	TIS-ma	WAL-fe	WAL-ma	LAR-fe	LAR-ma
LTR retrotransposons	Gypsy	Athila	17.54	18.79	18.18	18.53	18.37	17.90	18.08	18.61	18.00	18.31	18.37	17.88	16.90	17.09
		Ogre	17.10	16.61	17.22	15.53	16.28	16.11	16.96	16.80	15.93	14.54	15.87	15.00	16.67	15.21
		Chromovirus	11.28	12.86	12.63	12.45	12.75	12.90	12.17	13.62	12.19	11.94	12.44	12.54	12.13	10.94
		Tat	3.25	3.12	3.78	3.38	3.52	3.67	3.44	3.86	2.98	3.00	3.07	3.43	2.93	2.67
		sum	49.17	51.38	51.82	49.89	50.92	50.57	50.66	52.89	49.11	47.79	49.75	48.85	48.62	45.90
	Copia	Angela/Tork	16.51	16.09	16.18	16.92	16.36	16.45	16.92	15.88	18.08	17.91	17.97	18.61	17.65	19.49
		Maximus/SIRE	2.56	2.02	2.31	2.55	2.45	2.39	2.55	2.26	2.49	2.65	2.50	2.38	2.45	2.50
		TAR	0.22	0.32	0.20	0.23	0.24	0.22	0.19	0.22	0.21	0.23	0.24	0.25	0.29	0.22
		sum	19.29	18.42	18.69	19.71	19.05	19.07	19.66	18.36	20.78	20.80	20.72	21.24	20.39	22.22
Non-LTR retrotransposons	LINE		0.27	0.24	0.22	0.21	0.20	0.25	0.22	0.23	0.22	0.23	0.19	0.22	0.24	0.27
	Caulimoviridae		0.07	0.08	0.06	0.08	0.08	0.08	0.09	0.05	0.06	0.07	0.05	0.06	0.09	0.11
DNA transposons	CMC-EnSpm		1.70	1.63	1.66	1.67	1.67	1.70	1.74	1.63	1.69	1.69	1.80	1.75	1.76	1.76
	MULE-MuDR		1.65	1.33	1.45	1.56	1.48	1.50	1.52	1.51	1.44	1.55	1.48	1.54	1.19	1.42
	Helitron		0.18	0.20	0.16	0.19	0.16	0.18	0.16	0.16	0.18	0.19	0.18	0.19	0.25	0.23
		sum	3.23	3.16	3.27	3.42	3.31	3.38	3.42	3.30	3.30	3.44	3.46	3.48	3.41	3.41
Total TEs			71.69	72.96	73.78	73.02	73.28	73.02	73.74	74.54	73.19	72.03	73.92	73.57	72.21	71.53

Genome proportions of transposable element superfamilies and families in percentage for male and female from seven ecotypes

### Correlation between repeat abundance and genome size increase uncovered active repeats contributing to recent genome size variation

To identify recently active repeats, a correlation between repeat amount (obtained using RepeatExplorer tool) and genome size of both sexes was assessed across ecotypes. Figure 1c shows that most repeat types are positively correlated with genome size, but only some could be considered as statistically significant (marked with asterisks). This might reflect either different behavior of repeats in distinct ecotypes or conflicting effects of divergent lineages within respective repeat families. Therefore, the effect of particular LTR retrotransposon subfamilies was also assessed (Fig. 1d). The nine largest LTR retrotransposon subfamilies, previously classified in [21] were analyzed in detail. It was found that each subfamily has a specific behavioral pattern not necessarily identical to the whole family (Fig. 1c). Out of three Ogre subfamilies, OgreCL5 was found to be positively correlated while OgreCL11 was negatively correlated with the genome size (Fig. 1d). Overall, correlation analysis disclosed repeats influencing genome size variability across all ecotypes in a positive manner (AngelaCL1, AthilaCL3, OgreCL5, Caulimoviridae, and Helitrons) as well as in a negative manner (TekayCL4, OgreCL11). These repeats represent transpositionally active and silent TEs, respectively. Nevertheless, other TEs might also contribute to genome size variation but their activity differs in individual ecotypes. Another noteworthy finding is that correlation is not always similar for males and females as exemplified by AthilaCL3, OgreCL5, Chromoviruses and TAR elements showing positive correlation in females but lower or even negative correlation in males (Fig. 1c and d). This indicates higher insertional activity of mentioned TEs in the female genome (autosomes and X chromosomes), i.e. low insertional activity into Y chromosome. In contrast, only AngelaCL7 and minor TE families, LINE and Caulimoviridae, have higher insertional activity on the Y chromosome.

### Most of the retrotransposons are depleted on the Y chromosome

To assess the potential impact of individual LTR retrotransposon subfamilies on genome size, their copy number was estimated in all samples (Fig. 1e). The copy numbers were plotted against genome size to assess two key behavioral features of studied LTR retrotransposons; change of an LTR retrotransposon copy number towards bigger genomes (Fig. 1e, dashed lines), and relative abundance of a retrotransposon in males in comparison to females (Fig. 1e, solid colored lines). Due to a negligible genomic proportion of endogenous retroviruses and DNA transposons, only LTR retrotransposons were examined. Figure 1e shows scenarios of TEs behavior. Steeply increasing copy numbers of AngelaCL1, OgreCL5 and AthilaCL10 suggest that these LTR retrotransposons are main genome size drivers in most ecotypes (dashed lines). In contrast, TekayCL4, OgreCL6, and OgreCL11 show low or no insertional activity as implied from decreasing quantity of their genomic copies. However, most of the LTR retrotransposons show to some extent variable transposition in individual ecotypes.

**Fig. 2:**
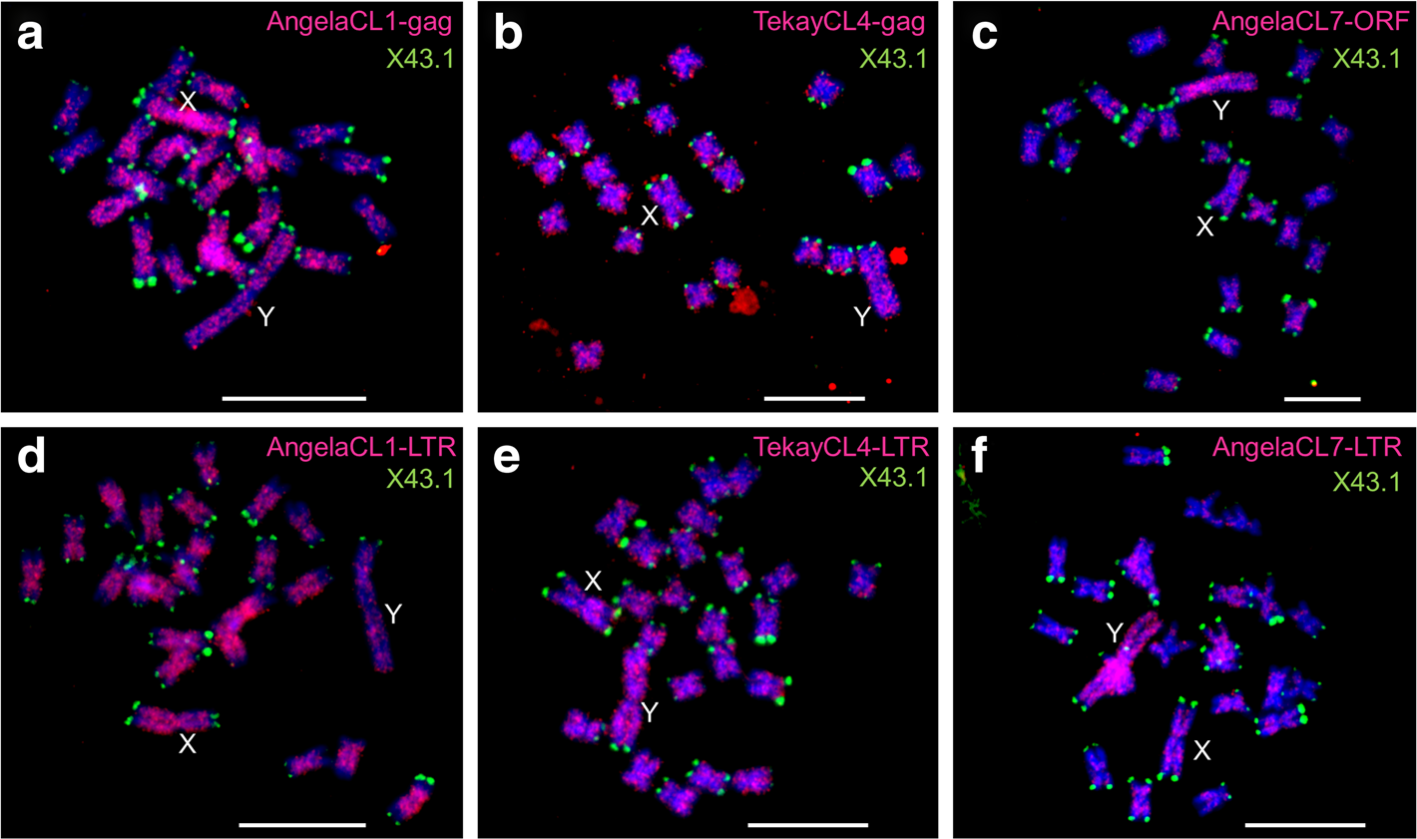
Localization of LTR retrotransposons on mitotic metaphase chromosomes of male *Silene latifolia* (Tišnov population) using fluorescence in situ hybridization (FISH). **a** AngelaCL1 gag and (**d**) LTR probe, (**b**) TekayCL4 gag and (**e**) LTR probe, (**c**) AngelaCL7 ORF and (**f**) LTR probe. Chromosomes were counterstained with DAPI (blue), LTR retrotransposon probes are represented by red signals, the tandem repeat X43.1 (green) labels most chromosomal subtelomeres, but only q-arm of the Y chromosome. Bars indicate 10 μm

Remarkably, most of the TEs differ in their abundance in male and female genomes (Fig. 1e, solid colored lines). Based on the fact that male genomes are ~ 4% larger than female genomes, slightly more TE copies are expected in males. However, most retrotransposons show even larger deviation from this expectation towards both directions. While some TEs are significantly more abundant in males (AngelaCL7, AthilaCL10), other TEs are significantly less abundant in male than female genome (AthilaCL3, OgreCL5). The former case indicates accumulation of TEs on the Y chromosome due to either reduced loss of DNA on the Y chromosome or higher activity of TEs in males. The latter case suggests the exact opposite; lower density of retrotransposon insertions on the Y chromosome than in the rest of the genome, which might be a consequence of either accelerated loss of DNA on the non-recombining Y chromosome or lower activity of retrotransposons in males. Unequal distribution of TEs on sex chromosomes assessed by a bioinformatics approach is in concordance with fluorescence in situ hybridization (FISH) experiments summarized in Table 2. For TEs with no published cytogenetic data available, we performed FISH on meiotic chromosomes of TIS ecotype (Fig. 2). Nevertheless, in specific cases, LTR retrotransposons differ in their behavior among ecotypes, as exemplified by AngelaCL1 which is underrepresented on Y chromosomes of all ecotypes except WAL and LAR (Fig. 1e (i)).

**Table 2:** Chromosomal distribution of retrotransposons with special emphasis on sex chromosomes revealed by fluorescence in situ hybridization (FISH) experiments

Subfamily	FISH	Citation
*Ty1/Copia*/AngelaCL1	Y-, X+	Fig. 2
*Ty3/Gypsy*/AthilaCL3	Y-, X+	Kralova et al., 2014
*Ty3/Gypsy*/TekayCL4	homogeneous	Fig. 2
*Ty3/Gypsy*/OgreCL5	Y-, X+	Kubat et al., 2014
*Ty3/Gypsy*/OgreCL6	Y- (slightly), X+ (slightly)	Kubat et al., 2014
*Ty1/Copia*/AngelaCL7	Y+, X-	Fig. 2
*Ty3/Gypsy*/RetandCL9	Y- (slightly), X+ (slightly)	Kejnovsky et al., 2006
*Ty3/Gypsy*/AthilaCL10	homogeneous	Kralova et al., 2014
*Ty3/Gypsy*/OgreCL11	homogeneous	Kubat et al., 2014

X+, Y+, the retrotransposon shows stronger hybridizing signal on the X and Y chromosome than on autosomes, respectively; X-, Y-, the retrotransposon shows weaker hybridizing signal on the X and Y chromosome in comparison to autosomes, respectively

To decipher the likely role of low Y diversity [30] in Y chromosome size constancy we constructed a copy number variability graph in male and female genomes (Additional file 4). The copy number values are adopted from Fig. 1e. The graph displays higher variability of TE copy numbers in males for the most abundant TE families. This additional copy number variability is driven by Y-linked TE copies and indicates that Y chromosome of each ecotype has unique repeat composition.

### The most active LTR retrotransposons preferentially proliferate in females

The conspicuous case among all repeats is LTR retrotransposon subfamily OgreCL5 which is virtually absent on the Y chromosome [8]. OgreCL5 is still an active element in all ecotypes as suggested by Fig. 1e (iv) and may be one of the dominant players in genome size variation among all *S. latifolia* ecotypes studied. An earlier publication proposed that OgreCL5 proliferates transgenerationally only in the female lineage [8]. This hypothesis was tested by estimating the density of OgreCL5 elements in X chromosomes in comparison with autosomes according to the formula *((F-M)/F) × 2/ 0.15* where *F* is a TE copy number in female (2C), *M* is a TE copy number in male (2C), and X chromosome accounts for 15% of genome length [9]. Since X chromosomes spend $$ \raisebox{1ex}{$2$}\!\left/ \!\raisebox{-1ex}{$3$}\right. $$ of their lifetime in females, while autosomes only $$ \raisebox{1ex}{$1$}\!\left/ \!\raisebox{-1ex}{$2$}\right. $$, the probability of insertion into the X chromosome for TE proliferating in females only is 1.33 times higher than into an autosome. In ecotypes LEL, TIS, WAL and LAR, X chromosome contains roughly 20–30% of all genomic OgreCL5 copies, 1.3–2 times more than an average autosome supporting the idea that OgreCL5 spreads preferentially in females and not in males. The computation is approximate due to the presence of a low but unknown number of OgreCL5 copies on the Y chromosome (mainly in pseudoautosomal region), thus it is slightly different from a theoretical value of 1.33. Because other retrotransposons with similar chromosomal pattern have even more Y-linked copies according to FISH experiments, the computation cannot be used for their copy number estimation – resulting copy number of X-linked TE copies would be undervalued in that case. Figure 1e and results of previous publications [4, 31, 32] examining the chromosomal localization of repeats (Table 2) suggest that at least *Ty3/Gypsy* LTR retrotransposons AthilaCL3, OgreCL6, and RetandCL9 also spread predominantly through female lineage but their recent retrotransposition activity is rather low in most ecotypes.

## Discussion

We have shown here that regardless of intraspecific genome size variation, the Y chromosome size is similar in European *S. latifolia* populations. Since *S. latifolia* is thought to have found refuge in North Africa during the last glaciations and to colonize its current range with the spread of agriculture [33, 34], the diversification of genome size is probably of recent origin. Unanswered questions remain: what is the ancestral state and what this variability of genomic sizes represents; are we observing rather expansion or reduction of genomes, or a combination of both phenomena here? If there is selective pressure to reduce the genome, there is no reason why X chromosome and autosomes should lose DNA faster than the largely heterochromatic (unpublished data) and genetically degrading non-recombining Y chromosome [35–38], which has lost 30% Y-linked genes [39, 40] and its diversity is reduced most likely due to strong selection against deleterious mutations [30]. Moreover, the genome of closely related *S. vulgaris* without sex-chromosomes is 2.7-fold smaller (see Plant DNA C-value Database, http://data.kew.org/cvalues/) indicating relatively recent genome expansion in *S. latifolia*. Thus, *S. latifolia* genome enlargement most probably continues as previously proven by [2] and also observed in other dioecious species [41], but at a various tempo in distinct populations. 1.07-fold variation in female genome size (Fig. 1a) indicates rapid genome size changes. And, importantly, the Y chromosome most likely contributes to genome size increase less than the rest of chromosomes.

This is in contradiction with existing assumptions that the evolutionarily recent Y chromosome (about 6 million years, [8]) is still in the expansion phase of evolution [1]. Extreme Y chromosome size [6, 42], gene degeneration [36, 43] and high content of repetitive sequences such as microsatellites [44], mobile elements and tandem repeats [4, 21, 45] and recent insertions of chloroplast DNA [46] as well as increased fixation of transposons on the Y chromosome in comparison to X and autosomes [47] illustrate the low efficiency of repair mechanisms requiring recombination.

The first possible explanation of almost constant Y chromosome size arises from low Y diversity [30, 35, 48, 49] caused most likely by selection against Y chromosomes with damaged essential genes [50] and by a selective sweep. Background selection and within-population hitch-hiking processes may lead to fixation of Y chromosomes with lower TE content that are now present across all populations. This is consistent with fixation of MITE copies on the Y chromosome of many European populations [47] and also with the fact that the Y chromosome effective population size is much smaller than that of X and autosomes [51, 52]. In this scenario, all Y chromosomes have to be homomorphic across populations not only on genic level but also in other sites as are in TE insertions. The latter condition is not met in case of *S. latifolia*. We constructed a copy number variability graph for TE families in male and female genomes (Additional file 4). The graph shows higher copy number variability of some TE families in male than female genomes across populations. The additional variability in male TE copy numbers is caused by TEs present on the Y chromosomes. This suggests that the Y chromosomes are polymorphic in TE composition, at least in case of the most abundant TE families. The genetic uniformity and reduced effective population size (at genic level) would be remnants of the last common ancestor, but in terms of TE content the Y chromosomes evolve independently since the subdivision of studied populations after the last glaciation.

The second hypothesis says that the slowdown of Y expansion is due to the increasing prevalence of deletion loss of non-recombining parts of the Y chromosome over the accumulation of repeats. This is consistent with massive loss of genes on the Y chromosome [39, 40]. Although this hypothesis seems to be likely, our data also favor an additional explanation that retrotransposons tend to spread more in the maternal line than in the paternal, resulting in a low frequency of insertions into the Y chromosome and its lack of growth over the rest of the genome. This phenomenon was initially observed by cytogenetic analyses when it was found that several LTR retrotransposons show a lower hybridization signal on the Y chromosome of *S. latifolia* [4, 8, 32, 53] and *R. acetosa* [5].

Whether the loss of DNA on the Y or male-specific silencing of TEs dominates is difficult to determine without comparisons of high quality reference genomes. Nevertheless, previous works confirmed that there is a number of active TEs in *Silene*, some of them with sex-specific mode of spread. For example, all Ogre elements, OgreCL5 absent on the Y chromosome as well as OgreCL6 and OgreCL11 present on the Y chromosome, peaked their retrotransposition activity after Y chromosome formation [8, 53]. This indicates rather male specific silencing of OgreCL5 than selective removal of this retrotransposon family from the Y. Several tens of thousands to 1 million years old TE insertions were also documented in X- and Y-linked BACs [45]. Moreover, some retrotransposons, especially *Ty1/Copia* group (AngelaCL7), recently accumulated on the Y chromosome (Fig. 1d, e (vi); Fig. 2c, f; [4]). Altogether, these facts suggest simultaneous activity of both TE types: dominating LTR retrotransposons that do not insert into the Y chromosome as well as LTR retrotransposons that contribute to Y chromosome enlargement, but not sufficiently to keep pace with the rest of the genome. Thus, the restricted expansion of the Y chromosome is likely caused by combination of both factors: (i) insertion of active LTR retrotransposons apart from the Y chromosome and (ii) deletion loss of DNA that to some extent compensates for the activity of transposons incorporating to the Y chromosome.

As noted above, high-quality *S. latifolia* reference genome sequence should enable us to obtain more rigorous evidence for TE activity within certain chromosomal regions, such as TE insertions age, location, and copy number. Unfortunately, only not-enough representative partial sequencing data (e. g. BAC clones or partially reconstructed genic sequences) are available so far. Moreover, only very complete reference genome sequence with high-quality assembly of TE islands can address all questions regarding TE age distribution and copy number. Thus, we believe that our approach based on a combination of FISH and TE copy number estimation from whole genome sequencing datasets obtained from several populations is sufficient for the conclusions.

Our bioinformatics and FISH analyses show that LTR retrotransposons follow one of three behavioral patterns: (i) LTR retrotransposons of the first group spread equally in all chromosomes and are represented by TekayCL4. (ii) The second group spreads preferentially in a female genome, which is manifested by their lower proportion on the Y chromosome and higher proportion on the X chromosome compared to autosomes (as a consequence of X chromosome spending $$ \raisebox{1ex}{$2$}\!\left/ \!\raisebox{-1ex}{$3$}\right. $$ of its existence in females, but only $$ \raisebox{1ex}{$1$}\!\left/ \!\raisebox{-1ex}{$3$}\right. $$ in males). This group exhibits a large variability. There are elements almost totally missing on the Y chromosome as well as elements only slightly underrepresented on the Y chromosome. The group is represented mostly by *Ty3/Gypsy* LTR retrotransposons, for instance, AthilaCL3, OgreCL5, and RetandCL9. (iii) LTR retrotransposons of the third group accumulate on the Y chromosome and have a lower copy number on the X chromosome than on autosomes, they spread predominantly in males and are represented by two smaller LTR retrotransposon families, AngelaCL7 and AthilaCL10. A unique case is AngelaCL1, which is accumulated on X chromosomes of most ecotypes but reveals Y chromosome accumulation in the southern European Larzac ecotype. This indicates not negligible degree of freedom in how a TE behaves in certain genetic background. All three behavioral patterns are also observable in *R. acetosa* [5].

A major question is whether the sex-dependent retrotransposition is specific for dioecious plants, or it is a common feature of retrotransposons in angiosperms? The second closely related question that resonates is how can retrotransposons be active preferentially in either male or female genome? To our knowledge, only a few cases of sex-specific retrotransposition have been documented in model plants, so far. Activated LTR retrotransposons EVADE (EVD) expand only if transmitted through the paternal germline but are epigenetically suppressed in female flowers of *Arabidopsis thaliana* [54]. Such retrotransposon regulation would result in accumulation on the Y chromosome in the dioecious system with XY sex-chromosomes. In contrast, OgreCL5 LTR retrotransposons absent on the Y chromosome of dioecious *S. latifolia* were shown to be most probably silenced during pollen grain development also by the epigenetic mechanism [8]. It has been suggested that TEs take advantage of temporal lack of epigenetic silencing during plant gametogenesis for their transposition [55, 56] but plants possess defensive mechanisms based on siRNA production in companion cells of plant gametes [57–60]. Nevertheless, epigenetic regulation is in current view a complex array of mutually interconnected pathways sharing signal molecules (siRNAs, lncRNAs) as well as proteins and enzymes (reviewed in [61, 62]). Thus, the way of certain TE silencing might be strongly individualized, which results in diverse chromosomal distribution of TEs in dioecious plants.

Another extremely important factor influencing TE silencing and activity is its position in the genome: near a gene, within a gene, in a TE island or at the centromere core (reviewed in [63]). In maize, TEs located near genes are subject of intensive RNA directed *de-novo* DNA methylation (RdDM), while TEs in intergenic regions remain densely condensed and heterochromatinized and show very low transcriptional activity, siRNA production and association with RdDM [64–66]. Unlike *Arabidopsis*, in large plant genomes, the near-gene RdDM activity may be critical for creating a boundary that prevents the spread of open, active chromatin to adjacent transposons [67]. Thus, proximity to genes is a major factor inducing RdDM, regardless of transposon sequence or identity, and is more associated with DNA transposons that tend to insert near genes and with short low-copy number retrotransposons than with long high-copy number LTR retrotransposons [64–66]. Therefore, long high-copy number LTR retrotransposons, that play a dominant role in genome expansion, are not likely target of RdDM but rather post-transcriptionally silenced by other small RNA based mechanisms. Several recent publications suggest that male reproductive organs adopted unique epigenetic pathways that utilize micro RNAs and tRNAs for efficient post-transcriptional silencing of TEs in pollen grains [60, 68]. Particularly tRNAs derived small RNAs were proved to target mainly *Ty3/Gypsy* LTR retrotransposons, which are dominant TEs in dioecious plants. Thus, the male germline might possess a reinforced epigenetic barrier against TE transposition compared to egg cell. The male-specific silencing of highly active retrotransposons might be an adaptive mechanism to retain genes essential for haploid pollen tube growth. In dioecious species, it would slow down genetic degeneration of Y-linked genes in addition to haploid purifying selection previously confirmed in *S. latifolia* [50]. A growing body of evidence indicates that male and female gamete formation is accompanied with differently efficient TE silencing mechanisms, what leads to diversity of TE ability to proliferate preferentially through either male or female lineage and subsequently to sex-chromosome specific distribution of TEs.

## Conclusions

Taken together, based on a combination of genome size estimation, repetitive DNA assembly, and analysis at the population level, we show that Y chromosome expansion has already peaked in *S. latifolia*. Our data suggest that first stage of sex chromosome evolution accompanied with Y chromosome expansion might present a relatively short period in raise and fall of sex chromosomes, since *S. latifolia* Y chromosome, in contrast to the human Y chromosome, is only partially degenerated. For a more complex view, genetic and genomic analysis should be combined in future experiments.

## Additional files

Additional file 1: Map with highlighted geographical locations where samples of wild *S. latifolia* plants were collected. Google is acknowledged for providing the map under fair use principles. (JPEG 291 kb)

Additional file 2: Title: Information about analyzed data. **Table S1** Geographical locations of wild *S. latifolia* populations used in this study. **Table S2** Genome size of individual samples estimated by flow cytometry. **Table S3** Detailed information about sequencing libraries of individual samples. **Table S4** Number of preprocessed reads used in analyses. (XLSX 13 kb)

Additional file 3: Information about studied LTR retrotransposons. (XLSX 8 kb)

Additional file 4: Plot displaying copy number variability of individual LTR retrotransposons between male and female genome in studied ecotypes. Values are adopted from the Fig. 1e. If Y-linked TE copy number is fixed, the copy number variability has to be lower in males than females. Equal or higher variability in males is clear sign of TE copy number variability on Y chromosomes. The figure suggests that Y chromosomes from distinct populations are highly polymorphic in TE content. (PDF 42 kb)

Additional file 5: Primers used for fluorescent in situ hybridization (FISH). (XLSX 8 kb)

Additional file 6: Detailed description of methods. (DOCX 41 kb)

## Abbreviations

BAC: Bacterial artificial chromosome
CD-Search: Conserved domain search
DNA: Deoxyribonucleic acid
FISH: Fluorescence in situ hybridization
lncRNA: Long non-coding RNA
LTR: Long terminal repeat
ORF: Open reading frame
RdDM: RNA-directed DNA methylation
siRNA: Small interfering RNA
TE: Transposable element
tRNA: Transfer ribonucleic acid
