# Supplementary figures and images for: The slowdown of Y chromosome expansion in dioecious Silene latifolia due to DNA loss and male-specific silencing of retrotransposons
Source: BMC Genomics. 2018 Feb 20;19:153. doi: 10.1186/s12864-018-4547-7 (PMC5819184; doi:10.1186/s12864-018-4547-7)

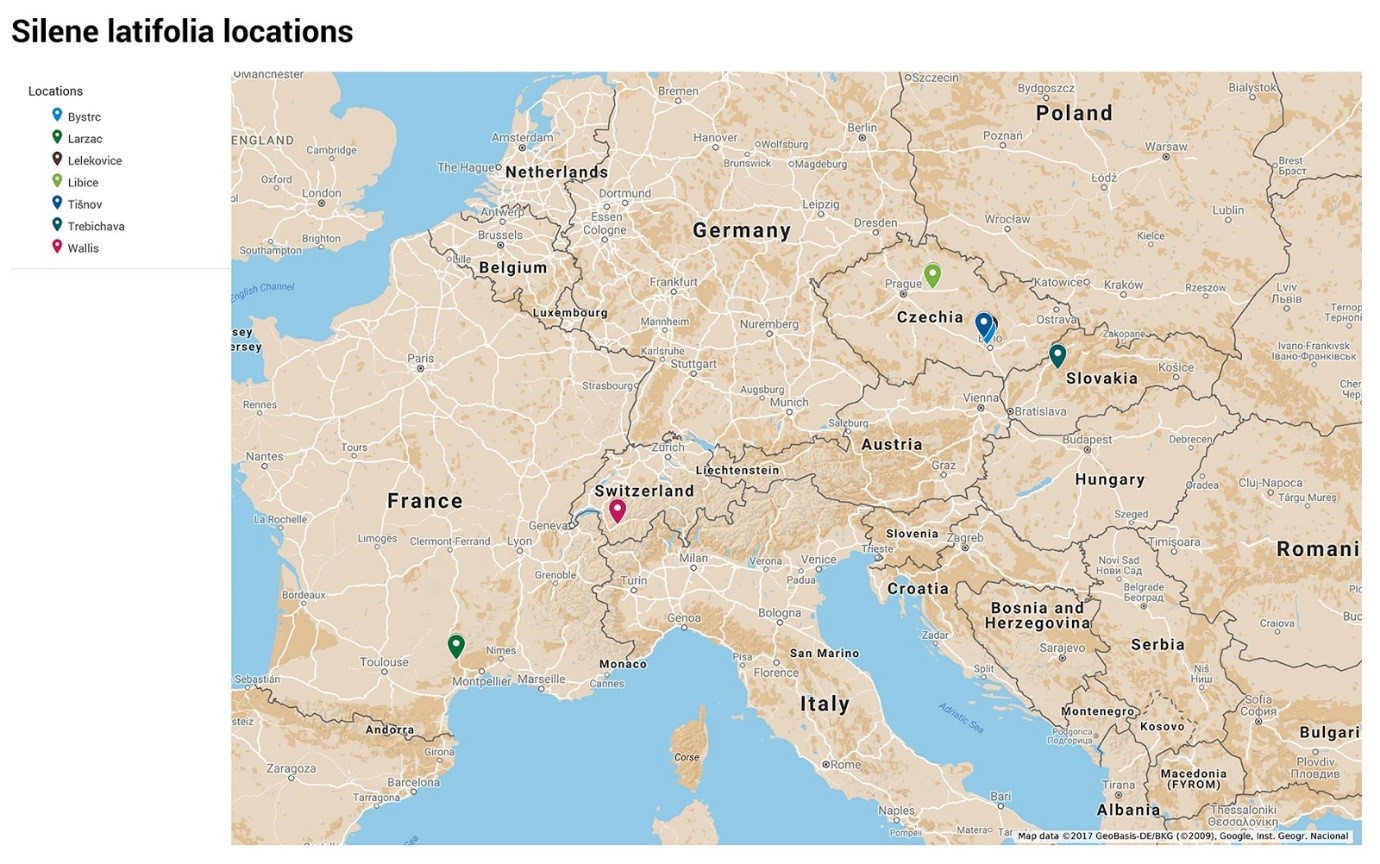

Supplement: Supplementary file 1 — Map with highlighted geographical locations where samples of wild S. latifolia plants were collected. Google is acknowledged for providing the map under fair use principles. (JPEG 291 kb) [file 12864_2018_4547_MOESM1_ESM.jpg]

Copy number

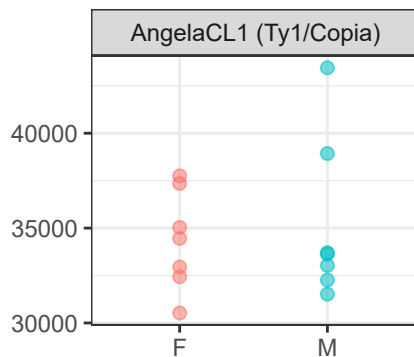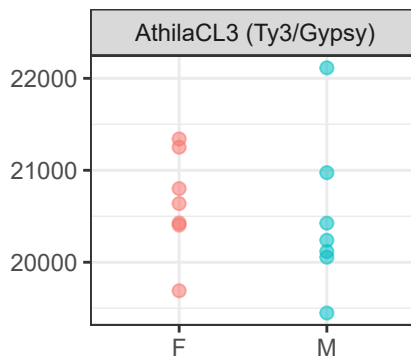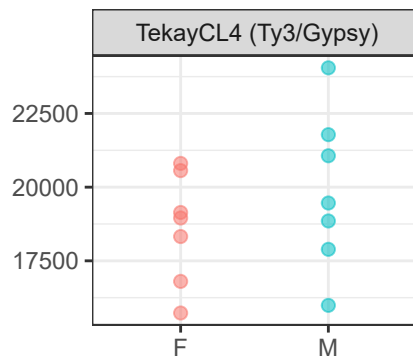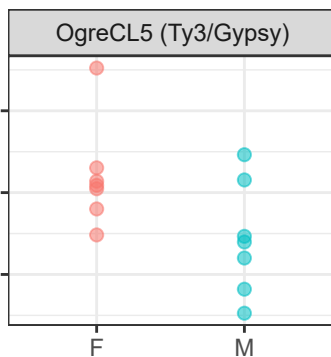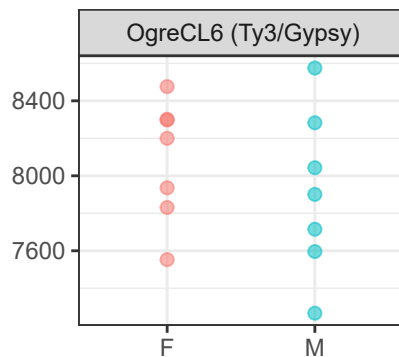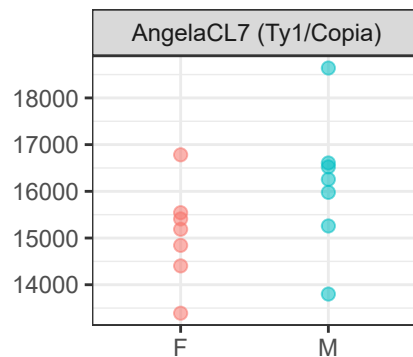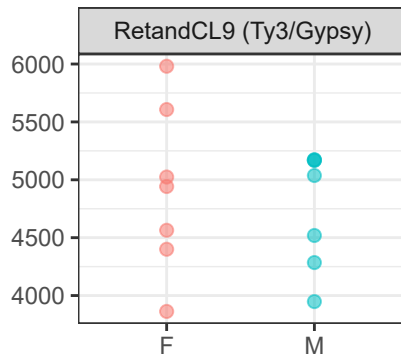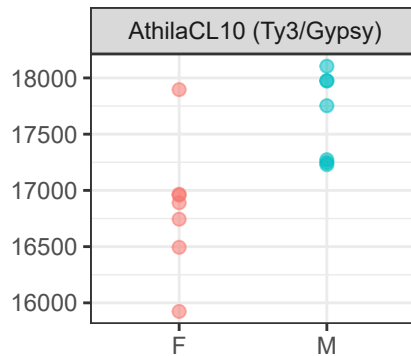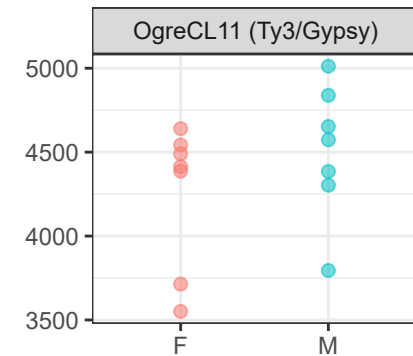

Sex

Supplement: Supplementary file 4 — Plot displaying copy number variability of individual LTR retrotransposons between male and female genome in studied ecotypes. Values are adopted from the Fig. 1e. If Y-linked TE copy number is fixed, the copy number variability has to be lower in males than females. Equal or higher variability in males is clear sign of TE copy number variability on Y chromosomes. The figure suggests that Y chromosomes from distinct populations are highly polymorphic in TE content. (PDF 42 kb) [file 12864_2018_4547_MOESM4_ESM.pdf]
