# Supplementary material for: The slowdown of Y chromosome expansion in dioecious Silene latifolia due to DNA loss and male-specific silencing of retrotransposons
Source: BMC Genomics. 2018 Feb 20;19:153. doi: 10.1186/s12864-018-4547-7 (PMC5819184; doi:10.1186/s12864-018-4547-7)
Supplement: Supplementary file 6 — Detailed description of methods. (DOCX 41 kb) [file 12864_2018_4547_MOESM6_ESM.docx]

**Flow cytometry and genome size estimation**

Approximately 30 mg of white campion leaf and 10 mg of soybean leaf (*Glycine max* L. cv. Polanka, 2C = 2.5 pg DNA), which served as the internal standard, were used for sample preparation. Suspensions of cell nuclei were prepared by simultaneous chopping of leaf tissues of white campion and soybean in a glass Petri dish containing 500 µl Otto I solution (0.1 M citric acid, 0.5% v/v Tween 20). Crude homogenate was filtered through a 50- µm nylon mesh. Nuclei were then pelleted (300 g, 5 min) and resuspended in 300 µl Otto I solution. After 30 min incubation at room temperature, 900 µl Otto II solution (0.4 M Na_2_HPO_4_) [1] supplemented with 50 µg/ml RNase and 50 µg/ml propidium iodide was added. Samples were analyzed using a Partec PAS flow cytometer (Partec GmbH, Munster, Germany). At least 5000 nuclei were analyzed per sample. Three male and three female individuals were analyzed for each white campion accession, and each individual was measured three times on three different days. Nuclear DNA content was then calculated from individual measurements following the formula: 2C nuclear DNA content [pg] = sample G_1_ peak mean of white campion / G_1_ peak mean of soybean. Mean nuclear DNA content was then calculated for each plant. Genome size (2C value) was then determined considering 1 pg DNA is equal to 0.978 × 10^9^ bp [2] and genome size of individual samples is available in Supporting Information Table S2.

**Sequencing library preparations and genomic DNA sequencing**

Genomic DNA used for sequencing was extracted from isolated leaf nuclei and was isolated using DNeasy Plant Mini Kit (Qiagen) according to the manufacturer’s protocol. DNA library preparations and sequencing services were provided by Centre of Plant Structural and Functional Genomics, Institute of Experimental Botany, Olomouc-Holice, Czech Republic. DNA libraries were prepared using Nextera DNA Sample Preparation Kit. The  *S. latifolia* genomes were sequenced by Illumina Nextera MiSeq platform and using protocol generating paired-end reads with average length ranging from 293.37 to 297.92 bp and fragments (insert size) of length between 1000 and 1500 bp (for details see Supporting Information Table S3). The sequencing has led to libraries of size ranging from 820 482 to 3 114 716 paired-end reads representing approximately 0.22 - 0.66x genome coverage. Whole genome sequencing data are available in European Nucleotide Archive. Primary accession: PRJEB21194.

**NGS data preprocessing**

Raw paired-end DNA reads were checked for quality using FastQC v0.10.5 (Andrews 2010, available online at: http://www.bioinformatics.babraham.ac.uk/projects/fastqc) and filtered by Trimmomatic v0.32 [4], filtering settings were estimated based on the FastQC quality reports as follows: PE -phred33 ILLUMINACLIP:/software/trimmomatic-0.32/adapters/TruSeq3-PE.fa:2:30:10 LEADING:30 TRAILING:30 SLIDINGWINDOW:4:20 MINLEN:200 CROP:200. Read names were manipulated to add specific codes to distinguish individual datasets. Because of computational restrictions of RepeatExplorer tool [5,6], which was used for repeat identification and characterization and will be described in the following section, all 14 datasets were randomly sampled to represent approximately 0.015x/1C (the exact number of reads is shown in Supporting Information Table S4) and 3 479 090 reads were analyzed altogether.

**Repeat identification and characterization from NGS data**

*De novo* identification and characterization of repetitive elements in *S. latifolia* was performed by RepeatExplorer pipeline [5,6] running in comparative mode, when reads from all 14 samples were clustered in one run with following settings: Minimum overlap length for clustering: 110 (55% length of read), All sequence reads are paired: yes, Rename sequences: yes, Length of sample code: 6, [%] Cluster size threshold for detailed analysis: 0.005, RepeatMasker database: Viridiplantae, Use custom repeat database: yes, Include sequence filtering?: no, Minimal overlap for assembly: 80 (40% length of read). Briefly, RepeatExplorer is a computational pipeline for identification and characterization repetitive elements or their parts from NGS data. It performs all-to-all similarity comparison of reads and records all read pairs which similarity satisfy given thresholds. Subsequently, reads are clustered using graph-based clustering according to similarities into groups of mutually connected reads which represent individual repetitive elements following by graph construction, where nodes represent reads and nodes are connected with edges based on similarity. Finally, it automatically characterizes resulting clusters based on similarity searches against RepeatMasker libraries, user custom libraries, blastn and blastx [7]. RepeatExplorer also provides information about cluster connections via paired-end reads. Because it tends to split large repeats into several clusters, the output was manually curated and re-annotated based on the paired-end read information, if necessary. Since it has been difficult to annotate smaller clusters, which represent low-copy repeats, only clusters containing more than 1000 reads were considered in further analyses. Thus processed data were used to estimate the proportion of individual repetitive.

**LTR retrotransposons subfamilies reconstruction**

Reference sequences of main LTR retrotransposon subfamilies presenting in *S. latifolia* genome were collected using assembled contigs published in Macas *et al.* (2011), more specifically LTR retrotransposons *Ty1/Copia*/AngelaCL1, *Ty3/Gypsy*/AthilaCL3, *Ty3/Gypsy*/TekayCL4, *Ty3/Gypsy*/OgreCL5, *Ty3/Gypsy*/OgreCL6, *Ty1/Copia/*AngelaCL7, *Ty3/Gypsy*/OgreCL8, *Ty3/Gypsy*/RetandCL9, *Ty3/Gypsy*/AthilaCL10, *Ty3/Gypsy*/OgreCL11. Contigs of these LTR retrotransposons were used as queries for megablast [9] searches against nr/nt database with default settings.  Nine of ten LTR retrotransposons had significant hits (see Supporting Information Table S5). OgreCL8 showed significant similarity with OgreCL5 downstream to protein domains in eORFs and 3’LTR region, and so these were considered as one subfamily. AngelaCL1 had significant hits in unannotated sequences with GenBank: AB257588.1 and GenBank: AB771926.1 and TekayCL4 subfamily showed no hits. Therefore, contigs of LTR retrotransposons AngelaCL1 and TekayCL4 were searched for presence of protein domains using CD-Search [10] with default settings. Thus annotated contigs were used as queries to search for similarities against assembled *S. latifolia* bacterial artificial chromosomes (BACs) provided by Institute of Biophysics, Department of Plant Developmental Genetics, Brno, Czech Republic using Geneious 8.1.7 software (http://www.geneious.com, Kearse *et al.* 2012), with similarity threshold set to 80%. Vicinity of hits was investigated by dotplot integrated in Geneious to find LTRs and ends of LTRs were estimated manually based on conservative 5’TG..CA3’ motif and TSDs (target site duplications). Full-length or almost full-length copies were cut out from BACs and aligned using MAFFT v7.017 (Algorithm: G-INS-i, scoring matrix 100PAM/k=2, Gap open penalty 1.53) [12]. Consensus sequences were generated using majority rule from sequences having more than 90% similarity over the coding region and were deposited in GenBank under accession numbers MF490430, MF490431.

**LTR retrotransposon subfamilies abundance and copy number estimation**

To estimate approximate abundance and copy number of main LTR retrotransposon subfamilies in *S. latifolia*, genomic reads were uniquely mapped onto reference sequences of individual subfamilies using Bowtie 2 v2.3.0 [13]. Target site duplications (TSDs) were removed from reference sequences and in the case of subfamily OgreCL11, insertion long about 1.3 kb was removed based on multiple alignment of incomplete OgreCL11 copies isolated from BAC clones. To capture variability of LTR retrotransposon subfamilies, the default settings of Bowtie 2 were relaxed in order to map reads with more mismatches as follows: bowtie2 --local --very-sensitive-local -x "${REFERENCE%%.*}" -1 $READS_1 -2 $READS_2 -f --minins 0 --maxins 1500 -p 4 --mp 1,1 --ma 6 --quiet. Coverage of subfamilies was obtained by samtools tool [14] using bedcov utility and copy number for the whole genome was calculated using a formula: *(subfamily coverage [bp]/subfamily_length [bp])*(100/0.75*), where 0.75 represents 0.75% 1C coverage. Density of OgreCL5 subfamily in X chromosomes in comparison to autosomes was estimated according to formula *((F-M)/F)*2/0.15*, where *F* is a copy number of OgreCL5 subfamily in female (2n), *M* is a copy number of OgreCL5 subfamily in male (2n) and 0.15 accounts for genome length of X chromosome [15].

**Fluorescence *in situ* hybridization (FISH)**

To synchronize the germinating seeds of *S. latifolia*, the DNA polymerase inhibitor aphidicolin was used, and mitoses were then accumulated with oryzalin. Slides were prepared from root tips and treated as described in Lengerova *et al.* (2004) with slight modifications. Slides were analyzed using the Olympus AX1 microscope, and image analysis using ISIS software (Metasystems). Average representatives out of 15-25 analyzed high-quality mitotic figures have been used for publication. To differentiate the arms of the Y chromosome, a cytogenetic FISH marker X-43.1 accumulated at subtelomeric regions of the majority of chromosomes was used [16]. Probes were prepared from PCR products with primers (Supporting Information Table S6) and genomic DNA from Tišnov ecotype. Primers were designed on LTR and GAG or ORF region of selected LTR retrotransposons using Primer3 [17].

**Statistical analysis**

All statistical analyses were performed in R version 3.4.0. Details of n for each experiment and the particular statistical test used can be found in the figure legends. The Pearson correlation coefficient of relationship between *S. latifolia* males and females genome size and repeat families abundance (Figure 1C), genome size and abundance of individual LTR retrotransposon subfamilies (Figure 1D), genome size and copy number of individual LTR retrotransposons subfamilies (Figure 1E) was calculated in R using *cor* function. P-values were obtained from *cor.test* function and adjusted p-values were calculated using *p.adjust* function using BH method implemented in R. Correlation statistics are presented in the results and Figure 1C, D, E. P-values and adjusted p-values lower than 0.05 were considered as statistically significant and are marked in Figure 1C, D, E by asterisks and double asterisks, respectively. Results throughout manuscript are represented as mean ± SEM. The coefficient of determination (R squared) in Figure 1E equals to the square of the Pearson correlation coefficient.

**References:**

1. Otto F. DAPI staining of fixed cells for high-resolution flow cytometry of nuclear DNA. Methods Cell Biol. United States; 1990;33:105–10.

2. Dolezel J, Bartos J, Voglmayr H, Greilhuber J. Nuclear DNA content and genome size of trout and human. Cytometry Part A. 2003. 51A:127–8.

3. Andrews S. FastQC A Quality Control tool for High Throughput Sequence Data. http://www.bioinformatics.babraham.ac.uk/projects/fastqc/.

4. Bolger AM, Lohse M, Usadel B. Trimmomatic: A flexible trimmer for Illumina sequence data. Bioinformatics. 2014;30:2114–20.

5. Novák P, Neumann P, Macas J. Graph-based clustering and characterization of repetitive sequences in next-generation sequencing data. BMC Bioinformatics. 2010;11:378.

6. Novák P, Neumann P, Pech J, Steinhaisl J, Macas J. RepeatExplorer: a Galaxy-based web server for genome-wide characterization of eukaryotic repetitive elements from next-generation sequence reads. Bioinformatics. 2013;29:792–3.

7. Altschul SF, Gish W, Miller W, Myers EW, Lipman DJ. Basic local alignment search tool. J. Mol. Biol. 1990;215:403–10.

8. Macas J, Kejnovský E, Neumann P, Novák P, Koblížková A, Vyskot B. Next generation sequencing-based analysis of repetitive DNA in the model dioecious plant *Silene latifolia*. PLoS One. 2011;6:e27335.

9. Zhang Z, Schwartz S, Wagner L, Miller W. A greedy algorithm for aligning DNA sequences. J. Comput. Biol. 2000;7:203–14.

10. Marchler-Bauer A, Bryant SH. CD-Search: Protein domain annotations on the fly. Nucleic Acids Res. 2004;32:327–31.

11. Kearse M, Moir R, Wilson A, Stones-Havas S, Cheung M, Sturrock S, et al. Geneious Basic: An integrated and extendable desktop software platform for the organization and analysis of sequence data. Bioinformatics. 2012;28:1647–9.

12. Katoh K, Standley DM. MAFFT Multiple Sequence Alignment Software Version 7: Improvements in Performance and Usability. Mol. Biol. Evol. 2013;30:772–80.

13. Langmead B, Salzberg SL. Fast gapped-read alignment with Bowtie 2. Nat. Methods. 2012;9:357–9.

14. Li H, Handsaker B, Wysoker A, Fennell T, Ruan J, Homer N, et al. The Sequence Alignment/Map format and SAMtools. Bioinformatics. 2009;25:2078–9.

15. Lengerova M, Kejnovsky E, Hobza R, Macas J, Grant SR, Vyskot B. Multicolor FISH mapping of the dioecious model plant, *Silene latifolia*. Theor. Appl. Genet. 2004;108:1193–9.

16. Bůžek J, Koutníková H, Houben A, Říha K, Janoušek B, Široký J, et al. Isolation and characterization of X chromosome-derived DNA sequences from a dioecious plant *Melandrium album*. Chromosom. Res. 1997;5:57–65.

17. Rozen S, Skaletsky HJ. Primer3 on the WWW for general users and for biologist programmers. Methods Mol. Biol. 2000;132:365–86.
